# An Asynchronous Collision-Tolerant ACRDA Scheme Based on Satellite-Selection Collaboration-Beamforming for LEO Satellite IoT Networks

**DOI:** 10.3390/s23073549

**Published:** 2023-03-28

**Authors:** Tao Hong, Rui Liu, Ziwei Liu, Xiaojin Ding, Gengxin Zhang

**Affiliations:** School of Communications and Information Engineering, Nanjing University of Posts and Telecommunications, Nanjing 210003, China

**Keywords:** LEO satellite IoT networks, random access, collaboration-beamforming, satellite-selection, collision-tolerant

## Abstract

In this paper, an asynchronous collision-tolerant ACRDA scheme based on satellite-selection collaboration-beamforming (SC-ACRDA) is proposed to solve the avalanche effect caused by packet collision under random access (RA) high load in the low earth orbit (LEO) satellite Internet of Things (IoT) networks. A non-convex optimization problem is formulated to realize the satellite selection problem in multi-satellite collaboration-beamforming. To solve this problem, we employ the Charnes-Cooper transformation to transform a convex optimization problem. In addition, an iterative binary search algorithm is also designed to obtain the optimization parameter. Furthermore, we present a signal processing flow combined with ACRDA protocol and serial interference cancellation (SIC) to solve the packet collision problem effectively in the gateway station. Simulation results show that the proposed SC-ACRDA scheme can effectively solve the avalanche effect and improve the performance of the RA protocol in LEO satellite IoT networks compared with benchmark problems.

## 1. Introduction

IoT is one of the main research directions for future 5G wireless network applications and plays an important role in industrial asset monitoring, smart cities, logistics and environmental data sensing [1,2,3,4]. As the number of IoT terminals increases, the number of terminals that can be served by a single terrestrial access point is limited, making it difficult to achieve massive information transmission. At present, information transmission between IoT terminals and base stations mainly relies on local area networks such as terrestrial wireless networks and Wi-Fi, which require the construction of a large number of terrestrial base stations to support. However, when IoT is applied in mountainous, marine and desert areas, there is a conflict between service capacity and network QoS requirements, as terrestrial base stations are not suitable for construction and maintenance in these remote areas [5]. Satellite communication systems have become an important complementary network to meet the global coverage requirements of 5G IoT applications due to their wide coverage range [6]. Therefore, IoT networks based on LEO satellites such as OneWeb, Starlink and Telesat LEO satellite constellation development plans have become a research hotspot in the field of IoT [7,8].

In the current LEO satellite communication scenario, an LEO satellite can cover a large number of IoT terminals, which use RA to exchange information with the satellite. RA will make different terminals occupy the same channel resources during communication, resulting in a collision between signals. For packets without collision, the receiver can demodulate them directly. However, if a collision occurs, packets will be lost or retransmitted, which leads to a decrease in system communication capacity and an increase in delay. At the same time, due to the long propagation delay between satellites and terminals, it is difficult for cheap IoT terminals to achieve strict network time synchronization. Therefore, the RA protocol of the asynchronous system is more suitable for the LEO IoT scene. However, the throughput performance of traditional RA protocols such as Pure-ALOHA (P-ALOHA), S-ALOHA (S-ALOHA) and diversity slotted ALOHA (DSA) is poor and can only be used for scenarios with a small number of terminals [9,10,11]. In pure ALOHA protocol, as long as the user is ready to send the packet, it will be sent immediately without checking whether other terminals are active. This system is simple to implement and suitable for situations with light traffic loads. However, when the load increases, the possibility of devices accessing simultaneously greatly increases, which will cause a sharp deterioration in access performance. Therefore, P-ALOHA has a peak load of only 0.184. S-ALOHA improves on P-ALOHA by dividing time into slots equal to the length of packets to ensure that no other packets arrive during the slot in which a single packet begins sending. This mechanism is an effective measure to reduce collisions and avoid arbitrary sending of packets by terminals, increasing throughput performance to 0.368. DSA protocol is an improved version of the S-ALOHA protocol. Although terminal packet transmission randomness was limited by introducing time slots in S-ALOHA protocol and reducing collision probability between packets. However, in the actual system, packet collisions still occur frequently, especially when the number of terminals in the system is large. The packets with collisions need to be resent, which seriously increases the access delay of satellite terminals. To alleviate this phenomenon, the DSA protocol uses the idea of time diversity, where each terminal makes a copy of the packet it wants to send and then randomly selects two different time slots within a data frame to send it. Some improved ALOHA solutions, such as collision resolution diversity slotted ALOHA (CRDSA), irregular repetition slotted ALOHA (IRSA) and coded slotted ALOHA (CSA), have all increased the throughput of the system. However, they still struggle to meet performance requirements in scenarios with a large number of IoT terminals [12,13,14]. CRDSA introduces SIC on the basis of the DSA protocol to recover collided packets. In CRDSA, two copies of a packet contain identical valid data information and corresponding slot position information for each copy selected. After successfully receiving one copy of a packet, its copy slot position can be located and eliminated. CSA protocol introduces linear coding modules that divide packets into several fields for linear encoding before transmission. Receivers use linear decoding and SIC techniques to resolve collisions. Under the condition of dual copies, CRDSA protocol can achieve a throughput of 0.612. On the basis of CRDSA, IRSA adjusts the number of copies when a terminal sends a packet. It changes the mechanism in CRDSA, where terminals send a fixed number of copies to select the number of copies based on probability distribution. In addition, some protocols adopt spread spectrum technology to improve the system throughput, such as spread spectrum ALOHA (SSA) and enhanced spread spectrum ALOHA (E-SSA) [15,16]. Since the spreading code has a time-delay capture characteristic, as long as the arrival times of two packets are not absolutely identical, these packets can be considered quasi-orthogonal. If the structure of the uplink receivers in the base station is reasonably set up, its collided packets can be demodulated. However, collision resolution with spread spectrum technology largely depends on packet arrival times and also lacks the utilization of spatial resources. The asynchronous contention resolution diversity ALOHA (ACRDA) protocol introduces the concept of virtual time slots in a data frame based on the CRDSA, changing the protocol from synchronous to asynchronous [17]. Similar to CRDSA, ACRDA solves the problem of packet collision through time and/or power/carrier frequency diversity. Overall, the above-mentioned improvements are limited to the processing of a single receiver and have weak capabilities in handling packet collision. Therefore, some improved solutions have begun to study the diversity of resources in the space domain of IoT, using LEO as distributed beamforming nodes for collaboration to improve performance or reduce system power consumption [18,19]. The authors of [20] proposed a novel multi-satellite cooperative random access (MSC-RA) scheme, which utilized multi-satellite overlapping coverage conditions for the IoT terminals. An asynchronous cooperative ALOHA (ACA) scheme is proposed in the literature [21], where packets generated by IoT terminals are received by multiple receiving satellites to form spatial copies of the packet. The un-collided packets on one satellite are then used to resolve collisions, which exist on other satellites using SIC technology, thus improving the system channel utilization. This scheme does not require the terminal to send additional packets compared to traditional schemes that transmit time-domain or frequency-domain copies classes, which not only saves power but also reduces the overall load on the network. However, the ACA scheme resolves packet collision only if at least one of the spatial copies of packets from multiple satellites has not collided before the SIC technique can be used to iteratively recover more collided packets. If all the satellite’s spatial copies of packets collided, the “deadlock” phenomenon will occur in the processing of the gateway station. ACA solution will not be able to resolve the “deadlock” packets, thus reducing the benefits of spatial diversity. A scheme based on packet signal strength indicator and geographic location information is proposed in the literature [22] to solve packet preamble collisions in grant-free random access (GFRA). This scheme is based on the characteristic that the adjacent access points of the IoT terminals have stronger channel gain. By extracting the geographic location of the colliding packet signals, the K-means clustering algorithm is used to cluster the adjacent access points of the colliding terminal based on the shortest distance. According to the feature of large-scale fading, the packet signal in the access point of the adjacent cluster of the colliding IoT is stronger, while the power of the interfering packet signals in this cluster may be weaker due to long distance, so that the power difference between the packet signal of this collided terminal and the interfering terminal can be used to demodulate the collided packet. In practice, however, the large number of IoT terminals will allow for a wide variety of collisions so that packet collisions may be generated by adjacent terminals. Based on large-scale fading only is insufficient to solve the performance problems caused by collisions, and on the other hand, a strictly synchronous regime is difficult to implement in the IoT system. The literature [23] proposes a cooperative beamforming ALOHA scheme (CBA) based on LEO, but this scheme only considers the cooperation scenario of dual satellites. In the future, it will be possible for multi-satellite co-view in the giant LEO constellation. We find that for the demodulation of desired signals, the cooperation performance of LEO satellites with poor channel quality will be reduced.

In this paper, we consider a network composed of IoT terminals, LEO satellites and ground gateway stations. Adjacent satellites collaborate to address the problem of collision in uplink transmission packets within the common coverage area at the gateway station. Due to the large number of LEO satellites in the co-visibility area, an optimized selection is made for participating satellites to maximize collaboration gain and reduce system power consumption. An SC-ACRDA scheme is proposed that maximizes throughput by addressing packet collision avalanche problems in LEO IoT uplink transmissions.

The main contributions of this paper are as follows:

- When the signal transmitted by satellite is subjected to beamforming processing by the gateway station, the participation of some channel nodes with poor channel quality in collaboration processing will reduce system performance. To address this issue, we proposed a collaborative multi-satellite selection algorithm based on maximum SINR. The algorithm solves the satellite selection problem in multi-satellite cooperative beamforming by constructing and solving a non-convex optimization problem. In order to make the optimization problem solvable, we use the Charnes-Cooper transformation to convert the non-convex problem into a convex optimization problem with optimization parameters. The optimized parameter determines the number of satellites selected. For the selection of optimized parameters, we design an iterative binary search algorithm to obtain optimal values;
- Regarding the problem of packet collision caused by the massive concurrent access of IoT terminals in LEO IoT network traffic surge. Under the ACRDA retransmission mechanism, an asynchronous collision-tolerant ACRDA scheme based on satellite-selection collaboration-beamforming is proposed by combining the proposed algorithm and SIC technology. The collided packets can be successfully demodulated through the power difference generated by the collaborative multi-satellite selection algorithm based on the maximum SINR algorithm. The demodulated packets can be reused for SIC. Simulation results show that the proposed scheme effectively solves the deadlock problem of packet collision under medium to high loads and improves system throughput.

The rest of this paper is organized as follows. Section 2 describes the system model. In order to solve the multi-satellite selection problem, the corresponding algorithm is designed in Section 3. In Section 4, the SC-ACRDA scheme is designed, and its performance is deduced. In Section 5, simulations and analysis are presented. Section 6 concludes this paper.

## 2. System Model

**Figure 1:**
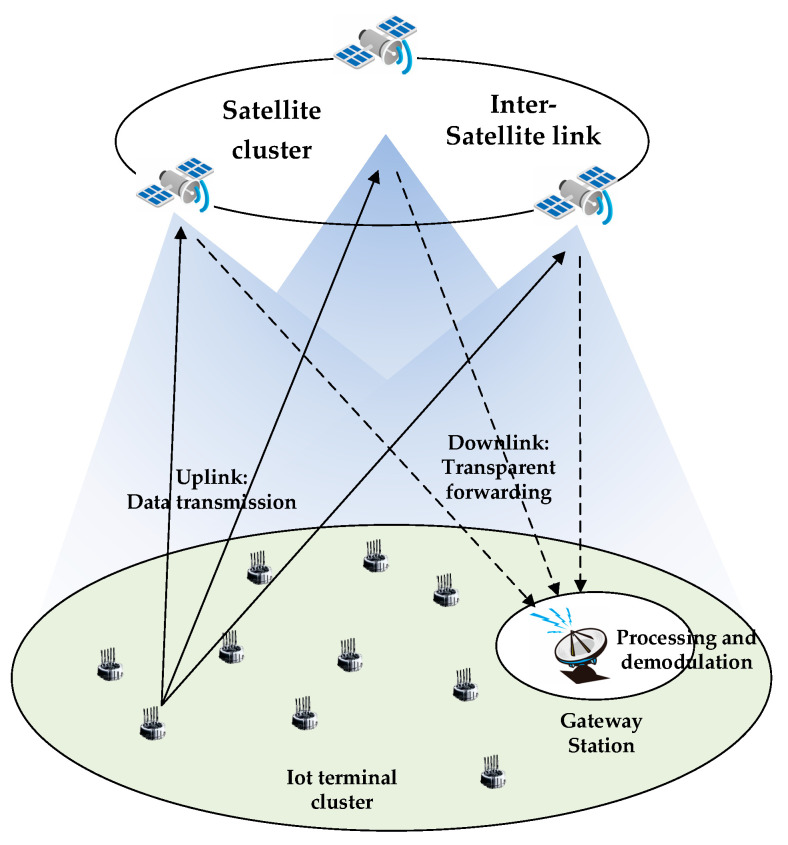
An IoT scenario models.

Consider a LEO satellite IoT scenario, as shown in Figure 1, which consists of multiple IoT terminals, a cluster of low-orbit satellites and a gateway station. When the service occurs, the terminal encapsulates the generated data into packets and transmits them to LEO satellites. The LEO satellite nodes are responsible for forwarding the information to the gateway station. The downlink transmission is in a transparent forwarding way, so we assume that there is no loss in the downlink. After receiving the forwarding signals, the ground gateway station will process and demodulate the signals to obtain the service information generated by the terminals. We assume a Rice channel between terminal and satellite, with the channel coefficients denoted by $h_{i}$, the second-order Reed–Muller (RM) sequence used to encode the preamble of packet. For packets without collision, the RM sequence carries the user ID information, so the gateway station can quickly detect the active user and perform channel estimation on the packet preamble to obtain the channel state information (CSI). For the collided packets, due to the nested structure of the subsequences of RM sequences, the corresponding CSI of the sequence can be obtained by the least squares (LS) channel estimation algorithm after the successful detection of the sequence according to the iterative RM sequence detection algorithm proposed in the literature [24]. At the same time, because the RM sequence has a larger sequence space when the same preamble length and a low detection complexity, the second-order RM sequence can also reduce the occurrence of packet collision phenomenon of the IoT. In terms of access protocols, the system uses the SC-ACRDA scheme proposed in this paper, where SC-ACRDA uses a multi-satellite selective collaborative beamforming algorithm to complement the elimination of collision scheme in the ACRDA protocol, as detailed in Section 4.

## 3. A Collaborative Multi-Satellite Selection Algorithm Based on Maximum SINR

In this section, we design a collaborative multi-satellite selection algorithm based on the maximum SINR to facilitate the gateway station to find the right satellites when collaborating on collided packets.

In the actual ultra-dense IoT network using RA protocol, uplink packet collision and retransmission lead to channel congestion, which limits the overall capacity of the system. When network traffic surges, lots of packets are transmitted at the same time, resulting in a collision between packets due to contention for channel resources. Collisions cause packet information to be lost, and the packet loss ratio (PLR) will increase as a result. In RA protocols, because of automatic repeat requests (ARQ), lost packets will be retransmitted within a certain period of time. If the channel is still under-resourced, the retransmitted packets will add to the congestion in the network. In addition, in some RA protocols that send copies, the lost packets will be retransmitted at multiplier levels, leading to an avalanche of degradation in system performance. Therefore, a collaborative approach to eliminating collision between packets in LEO satellite IoT is necessary. Although from an end-to-end performance perspective, full-node participation in collaboration is an optimal strategy. But in the actual collaboration process, adding satellite nodes with poor parameters to the collaboration will cause both instability in the processed SINR and additionally does not meet the low-power IoT foundation requirements. Therefore, inspired by the cluster selection scheme based on large-scale fading in the literature [22], this paper designed a multi-satellite selection collaboration algorithm based on maximum SINR to reduce system complexity and overall processing power consumption.

**Figure 2:**
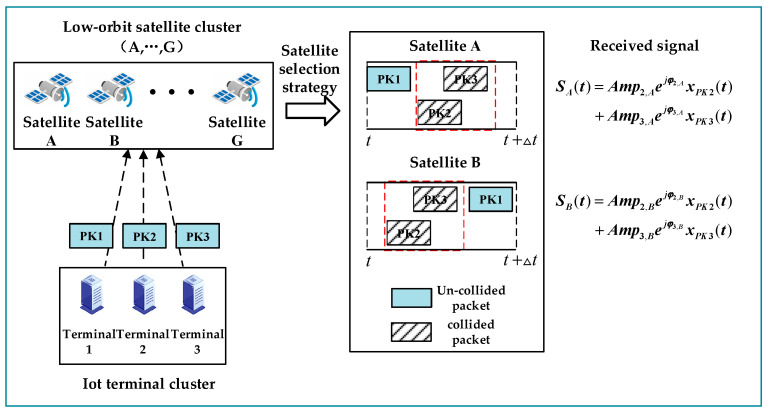
Multi-satellite selection algorithm application diagram.

To better illustrate the proposed scheme, consider a scenario, as shown in Figure 2, where the scenario consists of IoT terminals 1–3 and LEO satellites A–G. The terminal simultaneously sends packets PK1-3 to LEO satellites, assuming that PK2 and PK3 collide in all LEO satellites. The satellite is responsible for receiving and forwarding packet signals to the gateway station. At the gateway station, if a packet collision is detected in the forwarding signal of one satellite node, it searches for copies in other satellites with the same form of collision. Therefore, since the collision of PK2 packets is detected in satellite A–G, they can participate in processing as collaborative nodes. However, because of the difference in channel parameters, the collaboration of some nodes may reduce the processing gain. At this point, appropriate satellite collaboration nodes A and B can be obtained by algorithm from the forwarded signals of multiple collaborative satellites according to the satellite selection strategy. Subsequently, a distributed beamforming operation is performed between the selected nodes. The corresponding received signals in the nodes are multiplied by the optimal beamforming weights, which can make a power difference between two packet signals where the collision occurs. According to the idea of packet demodulation in the power domain in non-orthogonal multiple access (NOMA), if there is a large enough power difference between collided packets, then packets can be eliminated from collision and demodulated separately.

### 3.1. Proposed Satellite Selection Strategy

#### Construction of Optimization Problem for Maximizing SINR

**Figure 3:**
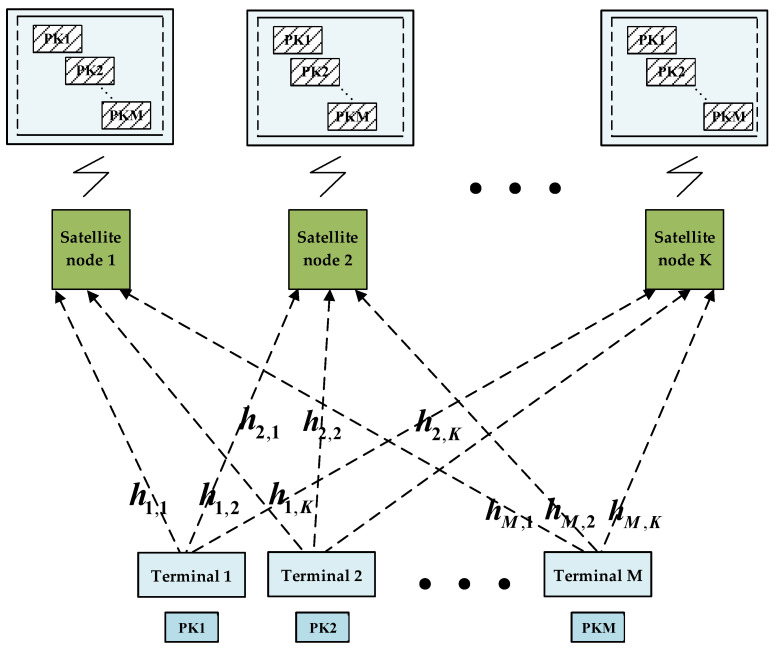
The relationship between terminals and satellite nodes when sending packets.

As shown in Figure 3, if packets from different terminals collide, all the satellite nodes covering these terminals are able to receive the collided packets. Our scheme aims to select the appropriate nodes from these satellites to collaborate and eliminate the collided packets. In the scenario shown above, where terminals 1–M send a packet at the same time, then in the RA protocol with no resource allocation, assume that all packets sent by M terminals collide at K satellite nodes. The signal received at a single satellite node can be modeled as follows:

(1) $$z_{i}=\sqrt{P_{t}}h_{1,i}S_{1}+\sum_{j=2}^{M}\sqrt{P_{t}}h_{j,i}S_{j}+n_{i}(1\leq i\leq K)$$

where $S_{1}$ is the expected signal vector, $S_{j},j\in [2,M]$ is the interference signal vector, $n_{i}$ is the noise signal component and $\sqrt{P_{t}}$ is the transmitting power. Beamforming is performed on the forwarded signal at the gateway station, and the receiver’s signal expression obtained is as follows:

(2) $$y=\sum_{i=1}^{K}\sqrt{P_{t}}w_{i}h_{1,i}S_{1}+\sum_{i=1}^{K}\sum_{j=2}^{M}\sqrt{P_{t}}w_{i}h_{j,i}S_{j}+\sum_{i=1}^{K}w_{i}n_{i}$$

Then, the useful signal power of the receiver can be expressed as follows:

(3) $$P_{S}=E\{|\sqrt{P_{t}}\sum_{i=1}^{K}w_{i}h_{1,i}S_{1}{|}^{2}\}=W^{H}AW$$

where $W={\left[w_{1},\ldots ,w_{K}\right]}^{H}$, $A=P_{t}E(H_{1}H_{1}{}^{H})$, $H_{1}={\left[h_{1,1},h_{1,2},\ldots ,h_{1,K}\right]}^{T}$. The power of interference and noise received at the receiver is as follows:

(4) $$P_{IN}=E\{|\sum_{i=1}^{K}\sum_{j=2}^{M}\sqrt{P_{t}}w_{i}h_{j,i}S_{j}{|}^{2}+|\sum_{i=1}^{K}w_{i}n_{i}{|}^{2}\}+\sigma_{d}^{2}=W^{H}BW+\sigma_{r}^{2}+\sigma_{d}^{2}$$

where $B=P_{t}E(H_{2}H_{2}{}^{H})$, $H_{2}={\left[h_{2,1},h_{2,2},\ldots ,h_{2,K}\right]}^{T}$, $\sigma_{r}^{2}$ is the noise variance between sender and satellites, and $\sigma_{d}^{2}$ is the noise variance between satellites and gateway station. Then the SINR of the receiver can be written as the following:

(5) $$SINR=\frac{P_{S}}{P_{IN}}=\frac{W^{H}AW}{W^{H}BW+\sigma_{r}^{2}+\sigma_{d}^{2}}$$

Assuming that the number of satellites expected to participate in the process is N. Selecting N suitable satellite nodes from K satellites, the non-convex optimization problem with Equation (5) as the objective function can be formulated as the following:

(6) $$\{\begin{array}{cc} \underset{W}{\max} & \frac{W^{H}AW}{W^{H}BW+\sigma_{r}^{2}+\sigma_{d}^{2}}\\ s.t. & \mathrm{card}(W)=N \end{array}$$

where card(W)=N represents the cardinality of the weight vector (the number of non-zero parameters in W). Moreover, when the gateway station processes the forwarded signal, a power processing upper limit needs to be set. The upper limit will make $|W{|}^{2}\leq 1$ and the individual component values $|w_{j}{|}^{2}$ as small as possible, ensuring the overall low energy consumption and physical realization possibilities of the system. It is assumed that the power processing upper limit for the selected j (1≤j≤N) forwarding signal is denoted $P_{j}$, which can be defined as follows:

(7) $$P_{j}=E\{|r_{j}{|}^{2}\}|w_{j}{|}^{2}=D_{j}|w_{j}{|}^{2}$$

(8) $$\begin{array}{ll} D= & [D_{1},\ldots ,D_{N}]=P_{t}diag(E\{|h_{1,1}{|}^{2}\}+E\{|h_{2,1}{|}^{2}\},\ldots ,\\ & E\{|h_{1,N}{|}^{2}\}+E\{|h_{2,N}{|}^{2}\})+\sigma_{r}^{2}I \end{array}$$

Therefore, optimization problems can be change to the following:

(9) $$\{\begin{array}{cc} \underset{W}{\max} & \frac{W^{H}AW}{W^{H}BW+\sigma_{r}^{2}+\sigma_{d}^{2}}\\ s.t. & \begin{array}{c} \begin{array}{cc} D_{j}|w_{j}{|}^{2}\leq P_{jth} & \forall j=1,\ldots ,K \end{array}\\ \mathrm{card}(W)=N \end{array} \end{array}$$

This problem is essentially an NP-hard problem, and according to the method proposed in the literature [25], the square of the norm $l_{1}$ can be used instead of the cardinality card(W). Because when setting the upper square limit parameter of $l_{1}$ norm, the sparsity of the corresponding vector (the number of 0 component) can be controlled. Therefore, the above optimization problem can be changed to the following:

(10) $$\{\begin{array}{cc} \underset{W}{\max} & \frac{W^{H}AW}{W^{H}BW+\sigma_{r}^{2}+\sigma_{d}^{2}}\\ s.t. & \begin{array}{c} \begin{array}{cc} D_{j}|w_{j}{|}^{2}\leq P_{jth} & \forall j=1,\ldots ,K \end{array}\\ ||W|{|}_{1}^{2}\leq \gamma \end{array} \end{array}$$

where $||W|{|}_{1}=\sum_{i=1}^{K}\left|w_{i}\right|$, γ is a set parameter. When γ changes, card(W) changes as well, and they are positively correlated. Since the direct use value vector W will affect the solvability of the optimization problem, matrix X can be defined as $X≜WW^{H}\in H_{N}$. X is the semi-positive definite Hermitian matrix of K×K, $||W|{|}_{1}^{2}={\left(\sum_{i=1}^{K}\left|w_{i}\right|\right)}^{2}={\left(1,\ldots ,1\right)}_{K}\cdot |X|\cdot {\left(\begin{array}{c} 1\\ \ldots \\ 1 \end{array}\right)}_{K}$, where |X| represents the absolute value of matrix elements. Then the above optimization problem can be changed to the following:

(11) $$\{\begin{array}{cc} \underset{W}{\max} & \frac{\mathrm{Tr}\{AX\}}{\mathrm{Tr}\{BX\}+\sigma_{r}^{2}+\sigma_{d}^{2}}\\ s.t. & \begin{array}{c} \begin{array}{cc} D_{j}|w_{j}{|}^{2}\leq P_{jth} & \forall j=1,\ldots ,K \end{array}\\ {\left(1,\ldots ,1\right)}_{K}\cdot |X|\cdot {\left(\begin{array}{c} 1\\ \ldots \\ 1 \end{array}\right)}_{K}\leq \gamma \\ X≽0\\ \mathrm{rank}(X)=1 \end{array} \end{array}$$

In Equation (11), rank(X)=1 condition will destroy the convex property of the optimization problem. This constraint can be removed by relaxation transformation, and Charnes-Cooper transform [26,27] is introduced as follows: $\sigma_{sum}^{2}=\sigma_{r}^{2}+\sigma_{d}^{2}$, $z=\frac{1}{\mathrm{Tr}\{BX\}+\sigma_{sum}^{2}}$, $Y=\frac{X}{\mathrm{Tr}\{BX\}+\sigma_{sum}^{2}}=zX$. Then the above problems can be converted into the following convex optimization problems:

(12) $$\{\begin{array}{cc} \underset{Y,z}{\max} & \mathrm{Tr}\{AY\}\\ s.t. & \begin{array}{c} \begin{array}{cc} Y_{jj}\leq \frac{zP_{jth}}{D_{j}} & \forall j=1,\ldots ,K \end{array}\\ {\left(1,\ldots ,1\right)}_{K}\cdot |X|\cdot {\left(\begin{array}{c} 1\\ \ldots \\ 1 \end{array}\right)}_{K}\leq \gamma \\ \mathrm{Tr}\{BY\}+\sigma_{sum}^{2}z=1\\ \begin{array}{c} Y≽0\\ z≽0 \end{array} \end{array} \end{array}$$

where z is a positive real number and Y is a complex semi-positive definite Hermitian matrix of order K. When the value of parameter γ is determined, the optimization problem can be solved by the CVX toolbox in Matlab. For optimization problem (12), the literature [28] points out that parameter γ is positively correlated with card(W), so the value of γ determines the sparsity of the weight vector matrix. Increasing γ will increase the diagonal non-zero elements of Y matrix, thus increasing the satellite nodes participating in the collaboration. Therefore, the selection of the forward signal is actually the problem of the value of γ. In order to solve this optimization problem, we design an iterative binary search method for γ to obtain the optimal value. After setting the upper limit $\gamma_{\max}$ and lower limits $\gamma_{\min}$, the optimal value $\gamma_{best}$ can be obtained by iteration. The pseudo-code of the Algorithm 1 is as follows:
**Algorithm 1:** Iterative binary search method of parameter γStep 1: InitializationSet the desired number of collaboration nodes: N; Set initial γ upper limit $\gamma_{\max}=\sum_{i=1}^{K}\frac{NP_{th}}{D_{i}}$; Set initial γ lower limit $\gamma_{\min}=0$; Set the initial value $\gamma =(\gamma_{\max}+\gamma_{\min})/2$; Define num as the number of non-zero diagonal elements of matrix Y; Substitute γ into Equation (12), calculate matrix Y and count num. Step 2: Iterative selection of optimal γ(1) while (num≠N)   if num>N//γ greater than optimal value $\gamma_{best}$, so reduce the upper limit $\gamma_{\max}$    if ($|\gamma -\gamma_{\min}|<0.1$)      $\gamma_{\min}=\gamma_{\min}/2$;      $\gamma =(\gamma_{\max}+\gamma_{\min})/2$;     else      $\gamma_{\max}=\gamma$;       $\gamma =(\gamma_{\max}+\gamma_{\min})/2$;    end  if num<N//γ less than optimal value $\gamma_{best}$, so increase the lower limit $\gamma_{\min}$    if ($|\gamma_{\max}-\gamma |<0.1$)      $\gamma_{\max}=\gamma_{\max}/2$;      $\gamma =(\gamma_{\max}+\gamma_{\min})/2$;     else      $\gamma_{\min}=\gamma$;       $\gamma =(\gamma_{\max}+\gamma_{\min})/2$    end  end end (2) while (num=N), the optimal value $\gamma_{best}$ can be obtained. Substituting $\gamma =\gamma_{best}$ into the optimization problem Equation (12), the matrix $Y^{*}$ can be calculated.Step 3: Determining the optimal subset of collaborationsAfter obtaining matrix $Y^{*}$, count its diagonal non-zero element indices and determine the optimal collaborative subset $R=\{R_{1},R_{2},\ldots ,R_{N}\}\in \{1,2,\ldots K\}$.

It should be noted that this algorithm is only used to obtain the optimal parameter $\gamma_{best}$, the subset $R=\{R_{1},R_{2},\ldots ,R_{N}\}\in \{1,2,\ldots K\}$ of the optimal collaboration and make the constraint condition card(W)=N hold. The weight vector W obtained by directly substituting $\gamma_{best}$ into Equation (12) is not accurate because the variance constraint of the $l_{1}$ norm restricts the value of W. In order to compute the optimal weight vector $W^{*}$, this constraint and the subset of relays that do not participate in the collaboration must be removed from this problem. Thus, when the optimal collaborative subset $R=\{R_{1},R_{2},\ldots ,R_{N}\}$ is obtained, the optimal weight vector $W^{*}$ needs to be derived from the following equation:

(13) $$\{\begin{array}{cc} \underset{\overset{⌢}{Y},z}{\max} & \mathrm{Tr}\{A\overset{⌢}{Y}\}\\ s.t. & \begin{array}{c} \begin{array}{cc} {\overset{⌢}{Y}}_{jj}\leq \frac{zP_{jth}}{{\overset{⌢}{D}}_{j}} & \forall j=1,\ldots ,N \end{array}\\ \mathrm{Tr}\{B\overset{⌢}{Y}\}+\sigma_{sum}^{2}z=1\\ \overset{⌢}{Y}≽0\\ z≽0 \end{array} \end{array}$$

where both $\overset{⌢}{Y}$ and $\overset{⌢}{D}$ are matrices corresponding to the optimal subset $R=\{R_{1},R_{2},\ldots ,R_{N}\}$. The optimal $\overset{⌢}{Y}$ matrix can be obtained by CVX. Assuming that ${\overset{⌢}{Y}}^{*}$ is the optimal solution matrix and $z^{*}$ is the optimal value, then ${\overset{⌢}{X}}^{*}=\frac{{\overset{⌢}{Y}}^{*}}{z^{*}}$. The optimal weight vector $W^{*}$ can be solved by the following:

(14) $$W^{*}=v_{max}\sqrt{\lambda_{\max}}$$

where $\lambda_{\max}$ is the maximum eigenvalue of the ${\overset{⌢}{X}}^{*}$ matrix and $v_{max}$ is the eigenvector corresponding to $\lambda_{\max}$.

## 4. An Asynchronous Collision-Tolerant ACRDA Scheme Based on Satellite-Selection Collaboration-Beamforming

This section introduces the design of the SC-ACRDA scheme, the packet processing flow in SC-ACRDA and its performance analysis.

### 4.1. Design of SC-ACRDA

**Figure 4:**
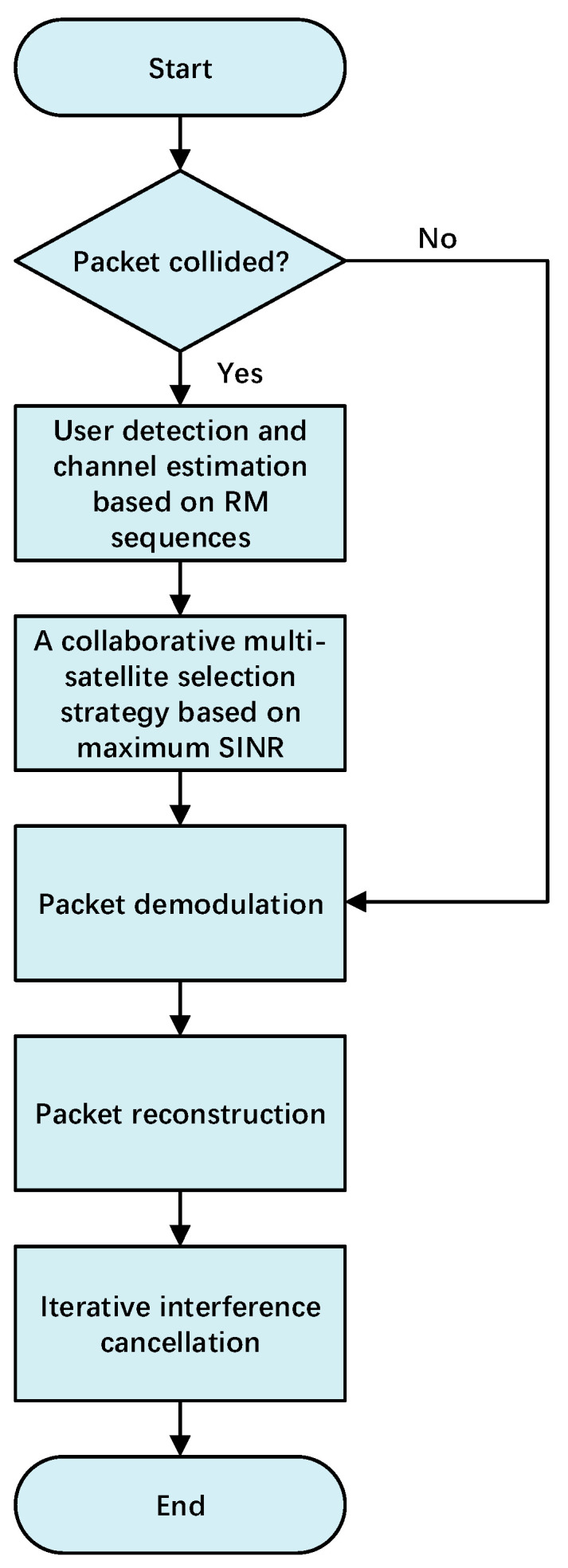
Basic flowchart of the SC-ACRDA scheme.

When a satellite node signal is forwarded to the gateway station, the gateway station will process each signal in parallel using multiple sliding windows. Figure 4 shows the basic flowchart of the SC-ACRDA scheme. For packets being processed:

A. If the packet did not collide:

(1) Packet demodulation: Obtain the RM sequences and demodulate the packet directly. In ACRDA protocol, the packet generates multiple time domain copies within a virtual frame. The packet preamble contains the slot offsets of the time domain copies. Therefore, after the packet has been demodulated, the location of copies can be found from the demodulated information.

(2) Packet reconstruction: Once the location of the copies is obtained, the packet in time domain can be reconstructed directly at the corresponding location of copies using the bit information of the already demodulated packet. For copies in the spatial domain in other signals, the gateway station performs an autocorrelation check of the RM sequence. If a correlation peak appears after the autocorrelation, the packet can be reconstructed at the location of the correlation peak. The packet reconstruction will eliminate the effect of the packet at the corresponding location.

(3) Iterative interference cancellation: When a packet copy is eliminated, new collision-free packets may be generated, so another round of iterations is performed to eliminate more packets until the maximum number of iterations.

B. If the packet collided:

(1) User detection and channel estimation based on RM sequences: For collision packets, due to the nested structure of the subsequences of second-order RM sequences, the matrix-vector pairs and CSI estimates of the collided packets can also be obtained by iterative RM sequence detection algorithms. CSI estimates can be further processed by LS channel estimators to obtain more accurate CSI. The matrix-vector pairs correspond one-to-one with the user information and can be used to determine whether the packet is a spatial domain copy.

(2) A collaborative multi-satellite selection algorithm based on maximum SINR: Once the packet signal identity information is obtained, the gateway station searches for the node where the packet collision occurred based on the identity information. All nodes obtained by searching can be collaborative node sets. The iterative binary search algorithm is performed on the set of collaborative nodes to obtain the optimal parameter γ. By substituting the optimal parameter γ into the proposed satellite selection algorithm, the optimal weight vector W can be obtained. The specific algorithm is given in Section 3. The corresponding node of the non-zero component of W is the set of optimal collaborative nodes. By using the optimal collaborative node set for distributed beamforming, the power difference can be generated between the collided packets. If the power difference is greater than the set threshold, the packet can be demodulated successfully. The processing process after successfully demodulating is the same as that of uncollided packets.

**Figure 5:**
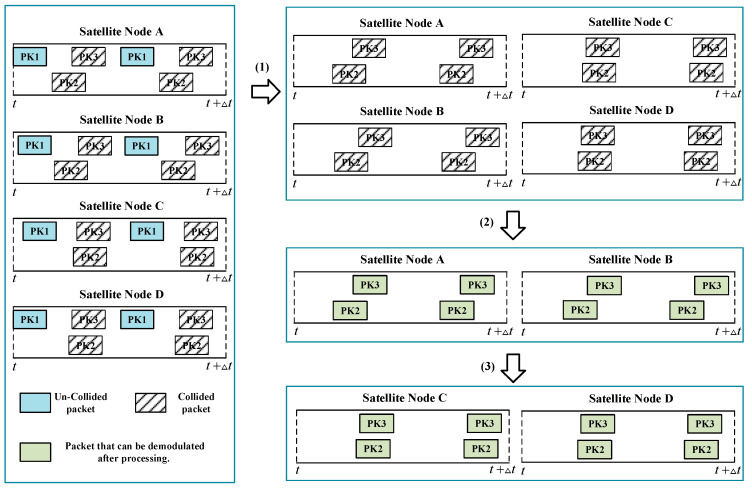
Packet processing flow chart.

To better illustrate the processing of packets, we give an example here. As shown in Figure 5, it is assumed that the gateway station receives four forwarding signals from satellites A–D. For these forward signals, since none of the first packets PK1 collided, they can be demodulated directly when processed. Moreover, subsequent copies of PK1 are eliminated as the preamble of PK1 contains the offset of its copy.

After (1) processing, only collided PK2 and PK3 remain in the forwarding signal. Since collisions also occur between copies of PK2 and PK3, these packets are deadlocked and cannot be processed according to the ACRDA demodulation strategy. As the preamble of PK2 in the forwarding signal can all be detected, the packet can be identified as PK2 based on the RM sequence characteristics. Satellites A–D all contain collided PK2, so they can be used as collaborative nodes for satellite selection.

The (2) processing is satellite selection and distributed beamforming. It is assumed that satellites A and B are the optimal collaborative set after selection, and packets meet the demodulation threshold after distributed beamforming. Therefore, PK2, PK3 and their copies will be able to be demodulated after (2) processing.

The (3) processing is the reconstruction of the packet and the elimination of copies. According to the processing in packet reconstruction, PK2 and PK3 can be used to demodulate and eliminate the corresponding copies in the forwarded signals of satellites C and D. Therefore, all packets in satellites C and D can be demodulated.

### 4.2. Performance Analysis

In this section, we will derive the PLR and throughput performance of this scheme based on ACRDA. For the ACRDA scheme, the packet loss ratio comes from the following two sources: (1) Packet loss due to the received packet SINR not reaching the threshold when no deadlock occurs; (2) packet loss due to the inability to handle collision when a deadlock occurs. Where a deadlock is a condition where all copies of a packet have collided (including the packet itself), this is known as a deadlock in ACRDA. Deadlock can take many forms on packets, from multi-packet partial collision deadlock to multi-packet full collision deadlock and so on, which makes it mathematically difficult to describe them directly. Therefore, in order to mathematically derive the PLR in the presence of deadlocks, the literature [29] considers limiting the analysis to the most basic and frequent deadlock approach to obtain a lower bound on the PLR. This basic deadlock approach is that the demodulated packet and its copies are expected to collide with only one packet. In the case of basic deadlock mode, the proposed scheme eliminates the collision that occurs between packets in some time slots. Therefore, the PLR of this scheme comes from the following: (a) packet loss due to the original ACRDA scheme where the bit SNR $E_{b}/N_{0}$ does not reach the demodulation threshold; (b) packet loss when the scheme cannot achieve sufficient power difference after collaborative beamforming is performed in the presence of deadlock. Assuming that the number of copies sent by the ACRDA scheme is $N_{cp}$, there are $N_{sl}$ time slots for a single data frame, and the number of iterative interference cancellations is $N_{iter}$. The total system packet loss ratio can be expressed as follows:

(15) $$\begin{array}{l} PLR^{Niter}(G,N_{cp},N_{sl})=PLR_{a}^{Niter}+PLR_{b}^{Niter}\\ \approx PLR^{Niter}(G,N_{cp})\cdot P_{dl}^{0}+\sum_{n=1}^{\infty }(1-\gamma )\cdot \Gamma {\left[10\log_{10}(\frac{\alpha }{1+\beta })\right]}^{N_{cp}}\cdot P_{dl}^{n} \end{array}$$

where $P_{dl}^{n}$ is the probability of the existence of n basic deadlock states, $P_{dl}^{0}\cdot PLR^{Niter}(G,N_{cp})$ is the PLR generated by iterating $N_{iter}$ times without deadlock, which is the PLR of case (a). $\sum_{n=1}^{\infty }(1-\zeta )\cdot \Gamma {\left[10\log_{10}(\frac{\alpha }{1+\beta })\right]}^{N_{cp}}\cdot P_{dl}^{n}$ is the PLR for case (b), where ζ is the statistical average success rate of relay collaborative beamforming in reaching the demodulation threshold. $\alpha =E\{{\left(E_{b}/N_{0}\right)}_{desire}\}$ is the mean value of the desired packet SNR. $\beta =E\{{\left(E_{b}/N_{0}\right)}_{in}\}$ is the mean value of the SNR of the interfering packets. Γ(x) is a polynomial interpolation of the packet error rate (PER) curve in dB for a given channel code and packet SNR x:

(16) $$\Gamma (x)=\{\begin{array}{cc} 10^{\left(\sum_{i=0}^{10}F_{i}x^{i}\right)} & x\geq T_{h}\\ 1 & x<T_{h} \end{array}$$

where $x=10\log_{10}(E_{b}/N_{0})$, $F_{0}=-0.29846$, $F_{1}=-0.53778$, $F_{2}=-0.23827$, $F_{3}=0.02605$, $F_{4}=-0.004$, $F_{5}=-0.01752$, $F_{6}=3.45\times 10^{-3}$, $F_{7}=2.02\times 10^{-3}$, $F_{8}=-3.52\times 10^{-4}$, $F_{9}=-3.46\times 10^{-5}$, $F_{10}=0$.

PLR for case (a):

For the ACRDA scheme, the PLR $PLR^{Niter}(G,N_{cp})$ after $N_{iter}$ iterations can be expressed as the cumulative integral of the probability density function $f_{d}(\alpha )$ of the expected packet SNR α multiplied by the expected PLR $P_{loss}^{Niter}{\left(\alpha ,\lambda \right)}^{N_{cp}}$ when the SNR is α and the Poisson service arrival rate is λ:

(17) $$PLR_{a}^{Niter}=PLR^{Niter}(G,N_{cp})\cdot P_{dl}^{0}$$

(18) $$PLR^{Niter}(G,N_{cp})\approx \int_{0}^{\infty }P_{loss}^{Niter}{\left(\alpha ,\lambda \right)}^{N_{cp}}\cdot f_{d}(\alpha )d\alpha$$

where, $P_{loss}^{Niter}(\alpha ,\lambda )$ can also be expressed as the cumulative sum of the loss rate $P_{loss}^{K,Niter}(\alpha |k)$ in the presence of k collided packets when the probability density function $f_{K}(k,\lambda )=\frac{\lambda^{k}e^{-\lambda }}{k!}$ of packet arrival and the SNR of the expected packet are α:

(19) $$P_{loss}^{Niter}(\alpha ,\lambda )\approx \sum_{k=0}^{\infty }P_{loss}^{K,Niter}(\alpha |k)\cdot f_{K}(k,\lambda )$$

Among them, the calculation of conditional probability $P_{loss}^{K,Niter}(\alpha |k)$ is relatively complex, which needs to consider the influence of the iterative interference elimination process, and its computational complexity increases exponentially. In the literature [17], the binomial distribution sorting method is considered to simplify the conditional probability without affecting the derivation of performance. The simplified formula can be expressed as follows:

(20) $$P_{loss}^{K,Niter}(\alpha |k)=\sum_{r=0}^{k}P_{loss}^{R}(r)\cdot f_{B}(r,k,q)$$

where $f_{B}(r,k,q)$ is the binomial distribution when the number of experiments is k and the success rate is $q={\left[PLR^{N_{iter}-1}(G,N_{cp})\right]}^{N_{cp}-1}$. The initial value of the packet loss ratio is 1: $PLR^{0}=1$. $P_{loss}^{R}(r)$ represents the loss rate when there is still r interference packets colliding with the expected packet after iteration $N_{iter}$, which can be expressed as follows:

(21) $$P_{loss}^{R}(r)=\int_{0}^{\infty }\Gamma [10\log_{10}(\frac{w}{1+\alpha x})]\cdot f_{\chi }(x,r)dx$$

where, $f_{\chi }(x,r)=\frac{1}{2\left(r-1\right)!}\sum_{n=0}^{r}{\left(-1\right)}^{n}\left(\begin{array}{l} r\\ n \end{array}\right){\left(x-n\right)}^{r-1}\cdot sign\left(x-n\right)$ is the probability density function when there are currently r packets and expected packets collided. x is the average number of packets collided with the expected packets. sign(●) is the indicating function.

PLR for case (b):

Assume that a virtual frame has $N_{sl}$ time slot and $N_{cp}$ packets are transmitted in this frame. If there is an interference packet in this $N_{sl}$ slot, then the total number of packets arranged in this frame is $C_{N_{slots}}^{N_{rep}}$. The probability of the interference packet colliding with the expected packet is $p=\frac{1}{C_{N_{slots}}^{N_{rep}}}$. Therefore, under the condition that the average number of packets arriving in each frame is $l=G\cdot N_{sl}\cdot \frac{1}{\rho \log_{2}M}$ (ρ is the channel coding rate, M is the modulation indices), $P_{dl}^{n}$ can be calculated as follows:

(22) $$P_{dl}^{n}={\left(1-p\right)}^{n}=\left(\begin{array}{c} l\\ n \end{array}\right)\cdot p^{n}\cdot {\left(1-p\right)}^{l-n}$$

When n≥2, the PLR performance is not affected due to the extremely low probability of $P_{dl}^{n}$ occurring. The PLR for case (b) can be simplified as follows:

(23) $$PLR_{b}^{N_{iter}}=(1-\zeta )\cdot \Gamma {\left[10\log_{10}(\frac{\alpha }{1+\beta })\right]}^{N_{cp}}\cdot P_{dl}^{n}+1\cdot (1-P_{dl}^{0}-P_{dl}^{1})$$

Thus, combining both cases (a) and (b), the total PLR of the SC-ACRDA scheme can be expressed as follows:

(24) $$\begin{array}{l} PLR^{Niter}(G,N_{cp},N_{sl})=PLR_{a}^{Niter}\cdot P_{dl}^{0}+PLR_{b}^{Niter}\\ \approx PLR^{Niter}(G,N_{cp})\cdot P_{dl}^{0}+(1-\zeta )\cdot \Gamma {\left[10\log_{10}(\frac{\alpha }{1+\beta })\right]}^{N_{cp}}\cdot P_{dl}^{1}\\ +1\cdot (1-P_{dl}^{0}-P_{dl}^{1}) \end{array}$$

Furthermore, for RA protocols, the throughput T(G) under a particular load G is directly related to PLR $PLR^{Niter}(G,N_{cp},N_{sl})$. The algorithm proposed in this paper consists of iterations and binary search, with a time complexity of approximately ${O(\mathrm{nlog}}_{2}n)$. Therefore, the throughput of this scheme can be expressed as follows:

(25) $$T(G)=G\cdot (1-PLR^{Niter}(G,N_{cp},N_{sl}))$$

## 5. Simulation and Analysis

In this section, we simulate the performance of the proposed multi-satellite selection algorithm based on maximum SINR and the performance of the SC-ACRDA scheme using this algorithm. A comparison is also made with the conventional scheme under the same conditions. The simulations are based on the Matlab platform and take the Monte-Carlo approach. In addition, the platform used in the experiment is AMD Ryzen 7 3700X-CPU@3.60GHz, GPU is RTX 2070S and memory is 16.00GB. The specific parameters of the simulation are shown in Table 1:

### 5.1. Performance of Multi-Satellite Selection Algorithm Based on Maximum SINR

**Figure 6:**
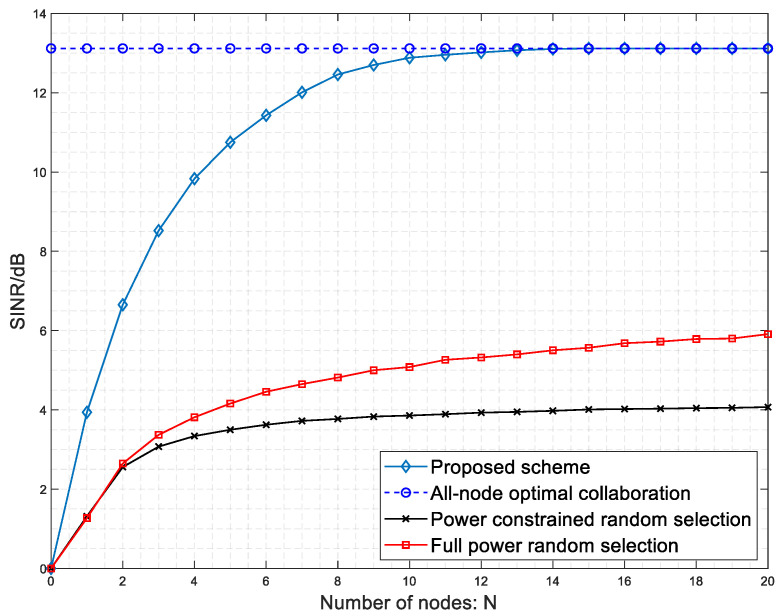
Comparison of SINR performance in four collaboration scenarios.

Figure 6 compares the SINR performance of the four collaboration scenarios. In Figure 6, the all-node optimal collaboration curve is the theoretical best performance for all forwarded signals selected and processed. The power-constrained random selection curve represents the performance obtained by randomly selecting nodes for beamforming of the forwarded signal under the processing power constraint. The full power random selection curve represents the performance obtained by selecting the maximum processing power for all nodes for the forwarding signal. The light blue diamond curve is the performance curve obtained by beamforming with node selection for this scheme. The graph shows that the proposed scheme improves the SINR of collided packets by a factor of 3–4 compared to the power-constrained random selection scheme. For example, with a node count of 10, the SINR when the proposed scheme is adopted is 12.73 dB, which is 3.25 times higher than the power-constrained random selection scheme with SINR = 3.91 dB. At the same time, a similar performance improvement to the power-constrained random selection scheme can be achieved at a much smaller power consumption compared to the full-power random selection scheme. Therefore, the proposed scheme can improve the SINR of the collided packet signal more effectively and increase the probability of the collided packet meeting the demodulation threshold than the random selection scheme. In addition, the proposed scheme consumes less power in the case of handling power constraints, making it more suitable for ultra-dense network scenarios where low power consumption is the norm.

### 5.2. Performance of SC-ACRDA

This section simulates and analyses the throughput and PLR curves for SC-ACRDA2 (packets with one-time domain copy), SC-ACRDA3 (packets with two-time domain copies) and other schemes with the corresponding number of copies (for schemes that do not send copies the corresponding number of receivers is increased).

**Figure 7:**
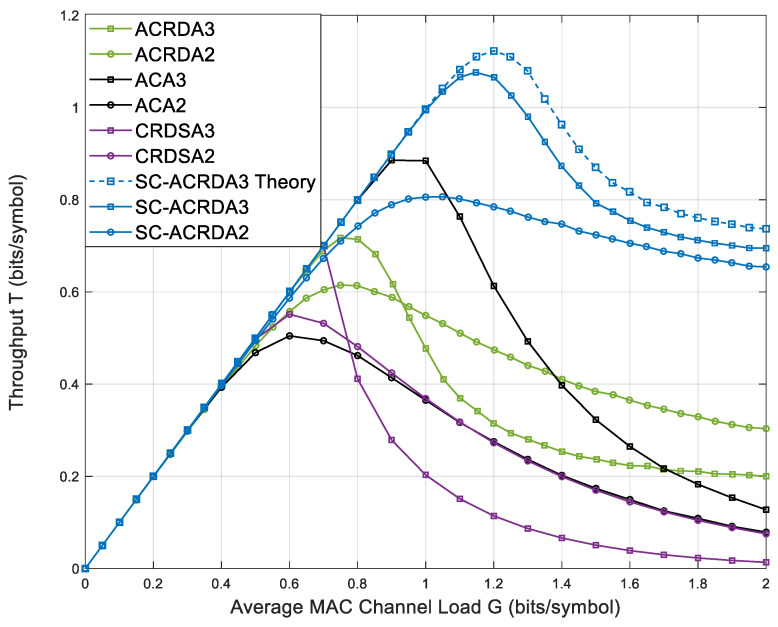
Comparison of throughput of SC-ACRDA2 and SC-ACRDA3 with other schemes.

Figure 7 shows the throughput comparison of SC-ACRDA2, SC-ACRDA3 and other benchmark schemes. Due to the multi-satellite selection beamforming algorithm, the expected SINR, which cannot reach the demodulation threshold in the original ACRDA scheme is increased. Enables some of these packets to be demodulated. After these packets are demodulated successfully, other packets that collide with the copies can also be demodulated due to the SIC mechanism. Thus, in theory, the SC-ACRDA2 scheme should be superior to the traditional RA scheme in both the peak throughput and high throughput load ranges (the throughput is greater than the load range of a specific value, which represents the packet processing capability of the scheme). Figure 7 shows that when the number of copies is 1, the peak throughput of SC-ACRDA2 reaches 0.806 when G = 1.05. ACA2 reaches a peak throughput of 0.505 when G = 0.6, ACRDA2 reaches a peak throughput of 0.614 when G = 0.75 and CRDSA2 reaches peak throughput of 0.552 when G = 0.6. When the number of copies is 2, SC-ACRDA3 reaches the peak throughput of 0.9939 when G = 1.40, ACA3 reaches the peak throughput of 0.886 when G = 1.0, ACRDA3 reaches the peak throughput of 0.7173 when G = 0.75 and CRDSA3 reaches the peak throughput of 0.700 when G = 0.7. Therefore, the peak throughput and high throughput load range of SC-ACRDA schemes are higher than those of traditional ACRDA schemes, regardless of one or two copies. The simulation curve is close to the theoretical curve, which verifies the correctness of the theoretical analysis.

**Figure 8:**
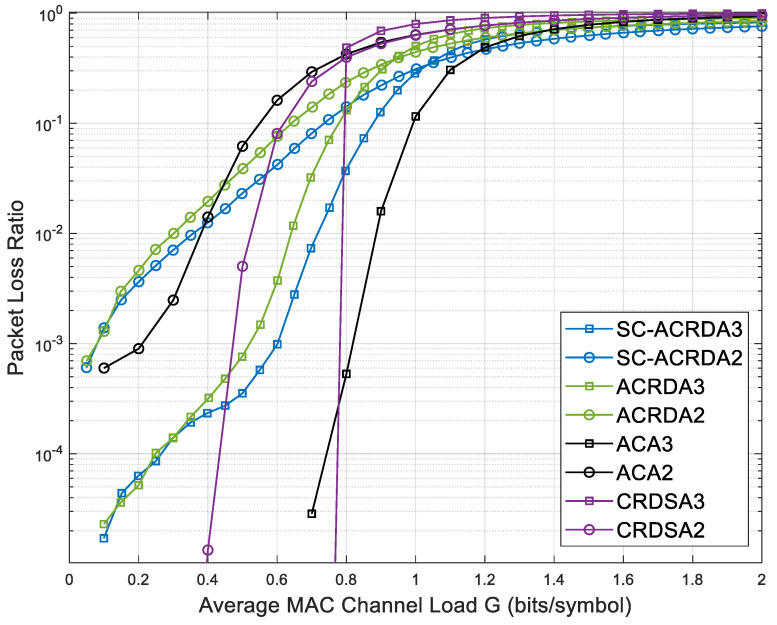
Comparison of PLR of SC-ACRDA2 and SC-ACRDA3 with other schemes.

Figure 8 shows the comparison of the PLR of SC-ACRDA2, SC-ACRDA3 and other schemes. At low load G = 0.1, the PLR is at level $10^{-3}$ (one copy) and $10^{-5}$ (two copies) because the packet arrival rate is low, and the packets nearly do not collide. When the load increases, the total number of packets in the virtual frame increases, the average number of packets allocated to each virtual frame slot increases, and non-demodulated collided packets are generated. As a result, the PLR gradually increases. Because the node selection algorithm is adopted, some collided packets can be demodulated. Therefore, the PLR of the SC-ACRDA algorithm is generally lower than that of the ACRDA algorithm at the load range [0.0, 1.2]. However, when the load continues to rise, the PLR of various algorithms will gradually converge. This is because when the total amount of packets is large, almost every time slot will collide, so only a few un-collided packets or a simple collision of two packets can be demodulated, and the PLR of various schemes will eventually tend to 1.

**Figure 9:**
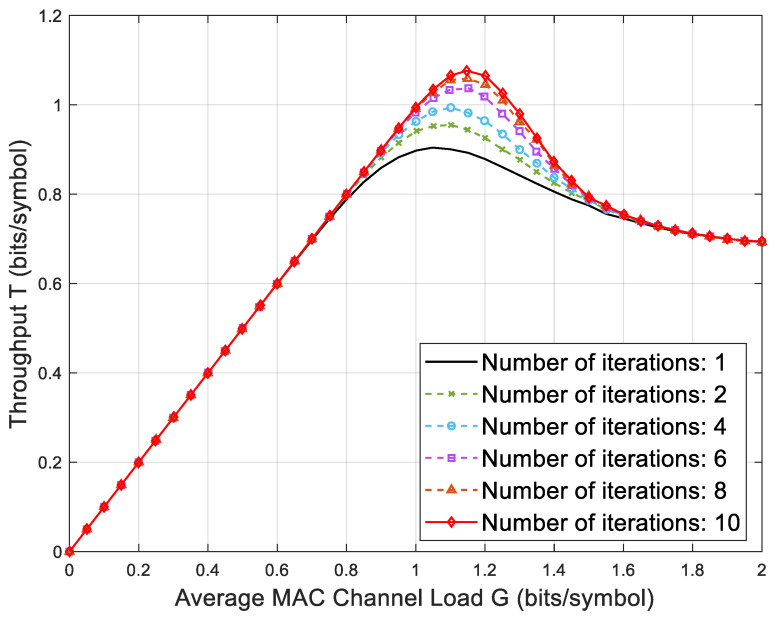
Throughput performance in relation to the number of iterations.

Since copies of packets can also be eliminated after demodulation, some collided packets may be demodulated when an iteration process is complete. Therefore, after the last packet is processed, the iteration will be repeated until no new packets are processed, so the throughput performance of the system is related to the number of iterations. Figure 9 shows the simulation diagram of the relationship between throughput and load under the condition that the number of copies of the SC-ACRDA scheme is N=2 and the maximum number of iterations is $N_{tier}=10$. It can be seen that when the load is less than 0.8, the throughput hardly improves with the increase in the number of iterations because of the small probability of packet collision. When the load is in the range [0.8, 1.4], two iterations bring a higher throughput benefit of about 0.10 bits/symbol, and the benefit of 6–10 iterations decreases gradually. When the load is greater than 1.6, iterative interference cancellation cannot be carried out due to widespread packet collision, and the system performance cannot be improved anymore.

## 6. Conclusions

In this paper, we study the RA protocol packet collision problem in LEO IoT. In order to improve the throughput performance of the RA system, an asynchronous collision-tolerant ACRDA scheme based on satellite-selection collaboration-beamforming is proposed to solve the collision problem of packets in a multi-satellite co-view scenario. Compared with the traditional full-node collaboration scheme, the proposed scheme can improve the power difference between collided packets more effectively with lower power consumption. The simulation results show that the proposed SC-ACRDA scheme can effectively improve the throughput performance of the RA protocol. The proposed solution is mainly used for the satellite selection problem in LEO satellite collaboration to avoid a collision, and it is also applicable to wireless communication systems that use unmanned aerial vehicles (UAVs) as relay transmission and adopt the RA protocol.

## Figures and Tables

**Table 1:** Simulation parameters.

Parameter	Value
LEO constellation	OneWeb
Maximum number of visible satellites	16
Number of transmitting sources	200
Number of virtual frames	2
Virtual frame length	100 slots
Packet arrival rate λ	[0.00:0.05:2.00]
Channel coding rate ρ	1/3
Modulation indices M	4
$\mathrm{Maximum}\;\mathrm{number}\;\mathrm{of}\;\mathrm{iterations}\;N_{iter}$	10
Collided packet demodulation threshold	7 dB

## Data Availability

Not applicable.
